# Supplementary material for: Getting More Out of Biomedical Documents with GATE's Full Lifecycle Open Source Text Analytics
Source: PLoS Comput Biol. 2013 Feb 7;9(2):e1002854. doi: 10.1371/journal.pcbi.1002854 (PMC3567135; doi:10.1371/journal.pcbi.1002854)
Supplement: Dataset S1 — GATE software. Dataset S1 bundled with this paper contains a distribution of GATE (or see http://gate.ac.uk/download/). (TGZ) [file pcbi.1002854.s001.tgz › plos-gate/src/gate/jape/zxc.html]

```
BasicPatternElement.java:  private JdmAnnotationSet matchedAnnots;
BasicPatternElement.java:  public JdmAnnotationSet getMatchedAnnots() { return matchedAnnots; }
BasicPatternElement.java:    matchedAnnots = new JdmAnnotationSet();
BasicPatternElement.java:    newPE.matchedAnnots = new JdmAnnotationSet();
BasicPatternElement.java:      JdmAttributeSequence newAttrs = newConstraint.getAttributeSeq();
BasicPatternElement.java:      JdmAttributeSequence existingAttrs =
BasicPatternElement.java:    matchedAnnots = new JdmAnnotationSet();
BasicPatternElement.java:    JdmDocument doc, int position, MutableInteger newPosition
BasicPatternElement.java:      JdmAttribute[] constraintAttrs = constraint.getAttributeArray();
BasicPatternElement.java:      JdmAnnotation match = doc.selectNextAnnotation(
BasicPatternElement.java:// moved to use array of JdmAttributes for selectNextAnnotation
Batch.java:  public void transduce(JdmCollection coll) throws JapeException {
Batch.java:      JdmDocument doc = coll.firstDocument();
Batch.java:    } catch(JdmException e) {
Batch.java:  public void transduce(JdmDocument doc) throws JapeException {
Batch.java:  public JdmDocument transduce(String text) throws JapeException {
Batch.java:    JdmDocument doc = new JdmDocument(text);
Batch.java:  public JdmDocument transduce(File textFile) throws JapeException {
Batch.java:  public JdmCollection transduce(String[] textFileNames) throws JapeException {
Batch.java:    JdmCollection coll = new JdmCollection(new JdmAttributeSequence());
Batch.java:    JdmDocument doc = null;
Batch.java:          new JdmAnnotationSet(),
Batch.java:          new JdmAttributeSequence(),
Batch.java:          JdmDocument.COPIED
Batch.java:      } catch(JdmException e) { throw new JapeException(e.toString()); }
Batch.java:    //gate.util.Debug.setDebug(JdmAnnotationSet.class, true);
Batch.java:    JdmCollection collection = null;
Batch.java:    JdmCollection coll = null;
Batch.java:        coll = new JdmCollection(persCollName);
Batch.java:      } catch(JdmException e) {
Batch.java:      try { coll.sync(); } catch(JdmException e) {
ComplexPatternElement.java:  public JdmAnnotationSet getMatchedAnnots() {
ComplexPatternElement.java:    JdmDocument doc, int position, MutableInteger newPosition
ComplexPatternElement.java:// moved to use array of JdmAttributes for selectNextAnnotation instead of a sequence
Constraint.java:    attrs1 = new JdmAttributeSequence();
Constraint.java:  public Constraint(String annotType, JdmAttributeSequence attrs) {
Constraint.java:    attrs1 = new JdmAttributeSequence();
Constraint.java:      attrs1.append((JdmAttribute) i.get());
Constraint.java:  private JdmAttributeSequence attrs1;
Constraint.java:  private JdmAttribute[] attrs2;
Constraint.java:  public JdmAttributeSequence getAttributeSeq() { return attrs1; }
Constraint.java:  public JdmAttribute[] getAttributeArray() { return attrs2; }
Constraint.java:  public void addAttribute(JdmAttribute attr) {
Constraint.java:    JdmAttribute attr = null;
Constraint.java:    // attribute constructor throws JdmException if the type of value
Constraint.java:      attr = new JdmAttribute(name, value);
Constraint.java:    } catch(JdmException e) {
Constraint.java:    newC.attrs1 = new JdmAttributeSequence();
Constraint.java:        newC.attrs1.append(new JdmAttribute((JdmAttribute) e.nextElement()));
Constraint.java:      } catch(JdmException ex) {
Constraint.java:      attrs2 = new JdmAttribute[0];
Constraint.java:    attrs2 = new JdmAttribute[attrsLen];
Constraint.java:      attrs2[i] = (JdmAttribute) e.nextElement();
Constraint.java://    	buf.append(" " + ((JdmAttribute) e.nextElement() ).toString());
Constraint.java:        buf.append(" " + ((JdmAttribute) e.nextElement() ).toString());
Constraint.java:// moved to use array of JdmAttributes for selectNextAnnotation instead of a sequence
ConstraintGroup.java:  public JdmAnnotationSet getMatchedAnnots() {
ConstraintGroup.java:    JdmAnnotationSet matchedAnnots = new JdmAnnotationSet();
ConstraintGroup.java:        JdmAnnotationSet patMatchedAnnots = pat.getMatchedAnnots();
ConstraintGroup.java:    JdmDocument doc, int position, MutableInteger newPosition
LeftHandSide.java:  public JdmAnnotationSet getBoundAnnots(String bindingName) {
LeftHandSide.java:  JdmAnnotationSet getMatchedAnnots() {
LeftHandSide.java:    JdmDocument doc, int position, MutableInteger newPosition
Matcher.java:    JdmDocument doc, int position, MutableInteger newPosition
MultiPhaseTransducer.java:  * Implements a transduce method taking a JdmDocument as input.
MultiPhaseTransducer.java:  public void transduce(JdmDocument doc) throws JapeException {
PatternElement.java:  abstract public JdmAnnotationSet getMatchedAnnots();
RhsAction.java:import gate.jdm.JdmDocument;
RhsAction.java:  public void doit(JdmDocument doc, LeftHandSide lhs) throws JapeException;
RightHandSide.java:      "  public void doit(JdmDocument doc, LeftHandSide lhs) { " + nl
RightHandSide.java:        "    JdmAnnotationSet " + name + "Annots = lhs.getBoundAnnots(\"" +
RightHandSide.java:  public void transduce(JdmDocument doc) throws JapeException {
Rule.java:  public int getNextMatch(JdmDocument doc, int position, int end) {
Rule.java:    JdmDocument doc, int position, MutableInteger newPosition
Rule.java:  public void transduce(JdmDocument doc) throws JapeException {
Rule.java:      doc.selectAnnotations("Name", new JdmAttributeSequence()).toString() +
SinglePhaseTransducer.java:  * Implements a transduce method taking a JdmDocument as input.
SinglePhaseTransducer.java:  public void transduce(JdmDocument doc) throws JapeException {
SinglePhaseTransducer.java:  protected void transduceBrillStyle(JdmDocument doc) throws JapeException {
SinglePhaseTransducer.java:  protected void transduceAppeltStyle(JdmDocument doc) throws JapeException {
SinglePhaseTransducer.java:// moved to use array of JdmAttributes for selectNextAnnotation instead of a sequence
TestJape.java:    JdmCollection coll = tokenise();
TestJape.java:      JdmAnnotation annot = coll.firstDocument().getAnnotations().nth(0);
TestJape.java:  "number", new JdmAttributeSequence()));
TestJape.java:  static public JdmCollection tokenise() {
TestJape.java:    JdmAttributeSequence cattrs = new JdmAttributeSequence();
TestJape.java:    JdmCollection collection = null;
TestJape.java:    JdmDocument testDoc1 = null;
TestJape.java:        collection = new JdmCollection(collectionName.getAbsolutePath());
TestJape.java:        collection = new JdmCollection(
TestJape.java:      JdmAnnotationSet cannot_set0 = new JdmAnnotationSet();
TestJape.java:    } catch(JdmException e) {
TestJape.java:  static public Transducer[] testParser(JdmCollection coll) {
TestJape.java:  public static void testBackEnd(JdmCollection coll) {
TestJape.java:    try { constraint.addAttribute(new JdmAttribute("kind", "otherNum"));
TestJape.java:    } catch(JdmException e) {
TestJape.java:    Transducer[] transducers, JdmCollection coll
TestJape.java:      } catch(JdmException e) {
TestJape.java:  static public void testCompilerOutput(JdmCollection coll) {
TestJape2.java:    //Debug.setDebug(JdmAnnotationSet.class, true);
TestJape2.java:    JdmCollection coll = tokAndGaz(collName, fileNames);
TestJape2.java:  static public JdmCollection tokAndGaz(String collName, Array fileNames) {
TestJape2.java:    JdmCollection collection = null;
TestJape2.java:        collection = new JdmCollection(collDir.getAbsolutePath());
TestJape2.java:      collection = new JdmCollection(
TestJape2.java:        collDir.getAbsolutePath(), new JdmAttributeSequence()
TestJape2.java:    } catch(JdmException e) {
TestJape2.java:      JdmAttributeSequence attrs = new JdmAttributeSequence();
TestJape2.java:      JdmDocument doc = null;
TestJape2.java:        JdmAnnotationSet annots = new JdmAnnotationSet();
TestJape2.java:      } catch(JdmException e) {
TestJape2.java:    Transducer transducer, JdmCollection coll
TestJape2.java:      JdmDocument doc = coll.firstDocument();
TestJape2.java:    } catch(JdmException e) {
Transducer.java:  public abstract void transduce(JdmDocument doc) throws JapeException;
```
